# Cuban medical training for South African students: a mixed methods study

**DOI:** 10.1186/s12909-019-1661-4

**Published:** 2019-06-17

**Authors:** Xincheng Sui, Priscilla Reddy, Anam Nyembezi, Pamela Naidoo, Kalipso Chalkidou, Neil Squires, Shah Ebrahim

**Affiliations:** 1 Department of Work and Social Psychology, Faculty of Psychology and Neuroscience, Maastricht University, Maastricht, Netherlands; 2 Population Health, Health Systems and Innovation, Human Sciences Research Council, Pretoria, South Africa; 3 Department of Social Work Faculty of Community and Health Sciences, University of the Western Cape, Cape Town, South Africa; 4 Global Health and Development, Institute for Global Health Innovation Imperial College London, London, UK; 5 Public Health England, London, UK; 6 London School of Hygiene & Tropical Medicine, London, UK

**Keywords:** Cuba, South Africa, Medical education, Mixed methods, Primary health care, Universal health coverage

## Abstract

**Background:**

Achieving universal health care coverage will require greater investment in primary health care, particularly in rural and underserved populations in low and middle-income countries. South Africa has invested in training black students from disadvantaged backgrounds in Cuba and large numbers of these Cuban-trained students are now returning for final year and internship training in South Africa. There is controversy about the scheme, the quality and relevance of training received and the place of Cuban-trained doctors in the health care system. Exploring the experiences of Cuban- and South African-trained students, recent graduates and medical school faculty may help understand and resolve the current controversy.

**Methods:**

Using a mixed methods approach, in-depth interviews and a focus group discussion were held with deans of medical schools, senior faculty, and Cuban-trained and South African-trained students and recent graduates. An online structured questionnaire, adapted from the USA medical student survey, was developed and administered to Cuban- and South African-trained students and recent graduates.

**Results:**

South African students trained in Cuba have had beneficial experiences which orientate them towards primary health care and prevention. Their subsequent training in South Africa is intended to fill skill gaps related to TB, HIV and major trauma. However this training is ad hoc and variable in duration and demoralizing for some students. Cuban-trained students have stronger aspirations than those trained in South Africa to work in rural and underserved communities from which many of them are drawn.

**Conclusion:**

Attempts to assimilate returning Cuban-trained students will require a reframing of the current negative narrative by focusing on positive aspects of their training, orientation towards primary care and public health, and their aspirations to work in rural and under-served urban areas. Cuban-trained doctors could be part of the solution to South Africa’s health workforce problems.

**Electronic supplementary material:**

The online version of this article (10.1186/s12909-019-1661-4) contains supplementary material, which is available to authorized users.

## Background

Reform in medical education has been spurred by growing health inequalities, changing demographic and epidemiologic patterns of disease, and massive disparities in access to health care [1]. Combating challenges of shortages and maldistribution of doctors, limited knowledge and imbalanced skill mix, and often, appalling work environments is critical [2]. Given the mobility of health workers, especially doctors, shared international responsibility for cooperative actions has been promoted [3, 4].

In low and middle-income countries all these challenges are more extreme. In South Africa, affirmative action policies in the eight medical schools have been implemented to allow black and women students from disadvantaged populations to enter medical school [5], which have increased the proportion of black students [6]. In 2014, of 9170 students in medical schools 39% were black, 33% white, 14% coloured and 14% Indian/Asian [6]. Despite increasing output from medical schools, the low ratio of doctors to population has not changed over the last decade due to population growth and migration of doctors [7–9].

Cuba’s approach to medical education has a strong focus on primary care, moving the centre of gravity from teaching hospitals to community facilities, and the promotion of polyclinics. Cuba has invested heavily in medical internationalism. At any time almost a third of Cuba’s 83,000 doctors are working outside Cuba, often in remote, underserved areas [10]. Cuba has also provided medical education for disadvantaged students from many countries, including the United States of America. Cuban medical education differs from conventional models by providing graduates with a wider skill set: care giver; decision maker; communicator; manager; community leader; teacher. The Cuban approach has potential in achieving Universal Health Coverage by training doctors who are able and willing to work in difficult situations [10].

A programme devised by Nelson Mandela and Fidel Castro to train disadvantaged black African medical students in Cuba was initiated in 1996, and was expanded in 2011, to meet the needs of rural and under-served urban areas. This expansion amounted to around 800 students per year training in Cuba with a total number of around 4000 training in Cuba by 2017 [11]. In 2018 around 700 Cuban-trained doctors returned to South Africa raising concern about how their training would be supervised and the subsequent costs of absorbing them into the health system [12]. The logic of training South Africans in another country, in a different language and culture has been criticized [8, 13]. As Cuban-trained graduates started to return to South Africa, further concerns were raised about their competences for practicing medicine. In response, South African medical schools have provided one to three years of extended medical school training on their return [14]. The situation has become politically polarised with South Africa’s opposition party claiming that money spent in Cuba would be better spent on expanding local medical education [15].

Health policy in South Africa is focused on Universal Health Coverage and in this context, training doctors with a strong primary health care orientation and a willingness to work in rural and urban under-served areas is essential. However, South African-trained doctors do not choose to make their careers in such places. It is estimated that only 35 of the 1200 students graduating annually from South African medical schools work long-term in rural areas [16].

Independent evidence on the effectiveness of the Cuban training programme is missing. A health technology assessment (HTA) approach (defined as “an intervention that may be used to promote health, to prevent, diagnose or treat acute or chronic disease, or for rehabilitation”) [17], treating medical education as a health technology was used to evaluate whether the Cuban training was fit for purpose. The following question was posed: how does the Cuban medical training in terms of perceived quality of teaching, competencies achieved, and career plans compare with South African medical training?

## Methods

A mixed-methods observational study of medical education in South Africa, comparing Cuban-trained and South African-trained students and graduates was conducted. Triangulation of findings from qualitative and quantitative studies was attempted to enhance their validity [18]. Field work was conducted by the Human Sciences Research Council of South Africa. The consolidated criteria for reporting qualitative research were used [19].

Access to medical schools was made by initial contact with Deans of Medical Schools after a prior stakeholder meeting held in 2015 in South Africa between government agencies, medical school faculty, Cuban-trained doctors, Human Sciences Research Council of South Africa and the research team [20]. Four South African medical schools participated in the study.

### Quantitative survey

A self-completed, online, structured questionnaire was developed to examine participants’ perceptions of the medical education they received, more specifically, appropriateness of training of medical science subjects, knowledge and skills acquired in talking to patients, relatives of patients and other professional team members, knowledge and skills acquired in clinical work, current ability to perform clinical skills without direct supervision, and career aspirations. Questions were drawn from the United States medical student survey questionnaires [21] which were validated by comparisons with medical education and medical care [22], and modified to cover specific topics relevant to South Africa (see Additional files 1 and 2). Face and content validity were considered by external experts in medical education. A convenience sample only of students and graduates (*n* = 71) was obtained as access to administrative databases was not permitted. Attitudes of participants were measured by 5-point Likert scales to gauge strength of views. Higher scores indicate higher levels of the construct measured.

Mann-Whitney U tests were conducted to examine the differences in attitudes between Cuban-trained participants and South African-trained participants. Due to multiple hypothesis testing, we applied Bonferroni’s correction to minimise type I error, with an adjusted α = 0.00094. In addition, Cronbach’s alpha statistics were calculated to assess the internal consistency of the items in each of the medical education domains (except for career aspirations as it is a multidimensional construct) and a mean score for each domain was calculated. Independent sample t-tests were conducted to examine the differences in these domains between Cuban-trained participants and South African-trained participants.

### Semi-structured interviews

Interviewers were conducted by two experienced senior investigators (PR, PN both women, see Additional file 3). It was not possible to draw participants from administration data bases so a convenience sample was obtained. Informed signed consent was obtained from interviewees and all interviews were tape-recorded and transcribed verbatim for thematic analysis. Themes were derived from the data and pre-existing themes arose from previous research and a stake-holder meeting held in South Africa [20]. Topics covered included quality of teaching and learning; competences of graduates; impact of graduates; potential and need for changes in curriculum, learning process, and evaluation. Interviewees comprised Deans of medical schools (*n* = 3), a medical student trained in South Africa (*n* = 1), a Cuban medical education programme co-ordinator (*n* = 1), and a senior lecturer (*n* = 1).

### Focus group discussion

The same interviewers conducted a group discussion among medical students in a South African medical school comprising 6th year students training in South Africa (*n* = 10) (see Additional file 4). Owing to continued student unrest on most university campuses during the field work it was not possible to gain access to other medical schools. Informed signed consent was obtained from participants. Similar themes were discussed as detailed above. The transcripts from the interviews and focus group discussions were analysed by a single investigator using themes that arose from the data and pre-existing themes that were derived from previous research and a stakeholder meeting [20]. Verbatim quotations were selected from the transcripts to illustrate the opinions and experiences of faculty members, students and recently qualified doctors.

Questionnaires and interview schedules are presented in the supplemental material.

## Results

### Quantitative findings

A total of 46 medical students completed the student survey (Cuban-trained students: *n* = 4; South African-trained students: *n* = 42). 25 Cuban-trained graduates who are currently practicing completed the medical health graduates survey (*n* = 25). The sample of Cuban graduates currently studying in South Africa (*n* = 4) and Cuban-trained graduates (*n* = 25) were combined (*n* = 29) to provide an overview of the experience of Cuban medical education for these participants. Responses of Cuban- and South African-trained participants were compared where feasible and with the caveat that these groups were a convenience sample at different points in their careers. Detailed descriptive data of Likert scale distributions of responses (numbers and percentages) are provided in Additional file 5.

There were marked differences in the demographic and social background of Cuban- vs. South African-trained participants. Cuban-trained participants were all black, 65% male, average age 34 years, 41% married, 14% had father with a university degree. By contrast, the South African-trained participants were 29% black, average age 24 years, 12% married, and 69% had a father with a university degree.

### Appropriateness of training

Reported quality of teaching in basic medical sciences at medical school showed marked differences between the groups (see Table 1). Cuban-trained participants rated most of the basic sciences as very good or excellent, particularly biochemistry, biostatistics and epidemiology, anatomy, social sciences, psychology, research ethics, and foreign languages.

**Table 1:** Experience of Basic Medical Science Topics

	Cuban-trained		South African-trained		Mann-Whitney Z	*p*
	Median	Mean (*SD*)	Median	Mean (*SD*)		
1. Biochemistry*	4.00	4.32(0.67)	3.00	3.36(0.91)	−4.29	.000
2. Biostatistics and epidemiology*	5.00	4.54(0.64)	3.00	3.10(1.09)	−5.34	.000
3. Genetics	4.00	4.04(0.88)	3.00	3.26(0.96)	−3.00	.003
4. Gross anatomy/dissection	5.00	4.32(0.98)	4.00	3.83(1.12)	−2.01	.044
5. Immunology	4.50	4.18(0.98)	4.00	3.74(1.04)	−1.83	.067
6. Introduction to clinical medicine	5.00	4.41(0.69)	4.00	4.17(0.89)	−0.98	.329
7. Social science, ethics, politics*	4.00	4.11(1.01)	3.00	3.07(1.26)	−3.32	.001
8. Microanatomy/Histology*	5.00	4.32(0.86)	3.00	3.05(1.29)	−4.07	.000
9. Microbiology	4.50	4.36(0.73)	4.00	3.62(1.08)	−2.96	.003
10. Neuroscience	4.00	3.78(0.89)	4.00	3.49(1.14)	−0.90	.368
11. Pathology	4.50	4.32(0.77)	4.00	3.86(1.03)	−1.88	.060
12. Pharmacology	4.00	4.29(0.71)	4.00	3.57(1.05)	−2.16	.031
13. Physiology	5.00	4.39(0.74)	4.00	3.91(1.05)	−1.98	.048
14. Psychology*	5.00	4.54(0.58)	3.00	3.21(1.26)	−4.52	.000
15. Pathophysiology of disease	4.50	4.39(0.69)	4.00	4.02(0.95)	−1.54	.123
16. Introduction to the patient	5.00	4.68(0.61)	4.00	4.10(1.04)	−2.61	.009
17. Research ethics*	4.00	4.29(0.81)	3.00	2.88(1.31)	−4.44	.000
18. Foreign languages*	5.00	4.36(0.87)	2.00	2.40(1.35)	−4.75	.000

*p < .00094

Note: The response scale has scores from 1 (very poor) to 5 (excellent), with an additional option for “not studied”

The participants that indicated a subject “not studied” were not included in the analyses for comparison

### Skills acquired

Knowledge and Skills Acquired in Talking to Patients, Relatives of Patients and other Professional Team Members.

Cuban-trained participants reported higher confidence than South African-trained participants in several clinical competencies (reflecting their more senior status), particularly, communicating appropriately in difficult circumstances and communicating health plans with local communities (see Table 2). The latter response is supported by over 69% of Cuban-trained participants experiencing multi-professional medical school education compared with 50% of those South African-trained.

**Table 2:** Knowledge and Skills Acquired in Talking to Patients, Relatives of Patients and other Professional Team Members

	Cuban-trained		South African-trained		Mann-Whitney Z	*p*
	Median	Mean (*SD*)	Median	Mean (*SD*)		
1. Elicit patients’ questions, their understanding of their condition and treatment options, and their views, concerns, values and preferences	5.00	4.59(0.50)	4.00	4.33(0.61)	−1.70	.089
2. Communicate clearly, sensitively and empathically with patients, relatives or other carers	5.00	4.59(0.57)	4.50	4.38(0.73)	−1.13	.257
3. Communicate appropriately in difficult circumstances*	4.00	4.41(0.57)	4.00	3.60(1.01)	−3.59	.000
4. Communicate health plans with local communities*	5.00	4.45(0.69)	4.00	3.69(0.92)	−3.67	.000
5. Know when to seek help from a senior colleague	5.00	4.55(0.51)	5.00	4.52(0.55)	−0.12	.903
6. Learn and work effectively within a multi-professional team	5.00	4.55(0.51)	4.00	4.19(0.67)	−2.30	.021

*p < .00094

Note: The response scale has scores from 1 (not all confident) to 5 (very confident)

### Knowledge and Skills Acquired in Clinical Work

Clinical work knowledge and skills were comparable between Cuban-trained participants and South African-trained participants (see Table 3).

**Table 3:** Knowledge and Skills Acquired in Clinical Work

	Cuban-trained		South African-trained		Mann-Whitney Z	*p*
	Median	Mean (*SD*)	Median	Mean (*SD*)		
1. Provide cardio-pulmonary resuscitation	4.00	4.14(0.88)	4.00	3.91(0.66)	−1.68	.094
2. Carry out practical procedures: venepuncture, taking blood cultures, measuring blood glucose	5.00	4.55(0.74)	5.00	4.88(0.33)	−2.35	.019
3. Establish peripheral intravenous access (set up an IV drip)	5.00	4.52(0.83)	5.00	4.62(0.66)	−0.36	.716
4. Carry out practical procedures: urinary catheterisation, skin suturing	5.00	4.45(0.83)	5.00	4.45(0.74)	−0.21	.831
5. Prescribe, set up and monitor a blood transfusion	5.00	4.14(1.06)	4.00	3.60(1.25)	−1.89	.058
6. Prescribe dose and route of insulin, including use of sliding scales	5.00	4.24(0.99)	3.50	3.41(1.08)	−3.24	.001

*p* < .00094

Note: The response scale has scores from 1 (not all confident) to 5 (very confident)

Current Ability to Perform Clinical Skills without Direct Supervision.

Cuban-trained participants reported higher confidence in their current clinical skills in conducting several tasks without supervision than South African-trained participants, including diagnose and manage acute medical emergencies, obstetrics, giving an anaesthetic for surgery, intubate and insert an endotracheal tube, and manage a primary health care team (see Table 4).

**Table 4:** Current Ability to Perform Clinical Skills without Direct Supervision

	Cuban-trained		South African-trained		Mann-Whitney Z	*p*
	Median	Mean (*SD*)	Median	Mean (*SD*)		
1. Diagnose and manage acute medical emergencies*	5.00	4.41(0.73)	4.00	3.36(0.98)	−4.60	.000
2. Obstetrics: carry out a forceps delivery*	4.00	3.90(1.32)	2.50	2.62(1.25)	−3.79	.000
3. Obstetrics: carry out a Caesarean section*	5.00	4.21(1.15)	2.00	2.17(1.12)	−5.53	.000
4. Give an anaesthetic for minor surgery*	5.00	4.45(0.74)	3.00	2.88(1.25)	−5.15	.000
5. Intubate and insert an endotracheal tube*	4.00	4.31(0.89)	4.00	3.45(1.02)	−3.73	.000
6. Give health promotion advice to mothers	5.00	4.66(0.55)	4.00	4.43(0.55)	−1.85	.065
7. Conduct a health survey in a local community	4.00	4.31(0.76)	4.00	4.00(0.91)	−1.42	.157
8. Manage a primary health care team*	5.00	4.55(0.63)	4.00	3.50(1.29)	−3.70	.000

* *p* < .00094

Note: The response scale has scores from 1 (not all confident) to 5 (very confident)

### Career aspirations

Motivations for becoming a doctor showed distinct differences (see Table 5). Cuban-trained participants reported stronger motivation for creativity and initiative in their career, to work in rural/under-served areas, to improve health of the country, and to become a community leader.

**Table 5:** Students’ Motivation for Their Choice of Medicine as a Career

	Cuban-trained		South African-trained		Mann-Whitney Z	*p*
	Median	Mean (*SD*)	Median	Mean (*SD*)		
1. Family wanted me to be a doctor	2.00	2.50(1.62)	2.00	2.12(1.19)	−0.75	.452
2. Good at sciences	4.00	4.07(0.75)	4.00	3.71(1.20)	−1.03	.303
3. Working for social change	5.00	4.31(1.00)	4.00	3.43(1.27)	−3.09	.002
4. High income potential	3.00	3.21(1.34)	4.00	3.50(1.11)	−0.80	.424
5. Desire to work in a rural/underserved area*	5.00	4.28(1.07)	2.00	2.36(1.14)	−5.46	.000
6. Desire to work in another country	1.00	2.03(1.27)	2.50	2.69(1.24)	−2.30	.022
7. Social recognition or status	1.00	1.96(1.32)	3.00	2.67(1.26)	−2.37	.018
8. Stable, secure future	4.00	3.79(1.24)	5.00	4.29(1.07)	−1.98	.048
9. Creativity and initiative*	4.00	4.10(0.86)	3.00	3.05(1.23)	−3.58	.000
10. Availability of jobs in the future	4.00	4.07(1.03)	5.00	4.33(1.03)	−1.53	.125
11. Work/life balance	4.00	4.00(1.13)	3.00	3.38(1.06)	−2.49	.013
12.Could not do my preferred subject/option	1.00	1.90(1.32)	1.00	1.45(1.09)	−1.74	.083
13. Desire to help other people	5.00	4.69(0.54)	4.00	4.07(1.07)	−2.69	.007
14. Improve health in my country*	5.00	4.69(0.54)	4.00	3.76(1.16)	−3.76	.000
15. Become a community leader*	5.00	4.35(0.97)	3.00	3.02(1.26)	−4.35	.000

* *p* < .00094

Note: The response scale has scores from 1 (not important at all) to 5 (extremely important)

The majority (80%) of the Cuban-trained participants indicated that they plan to work in primary health care (mostly in rural areas), whereas South African-trained participants (42.9%) reported that they do not plan to work in primary health care and only 20% wanted to work in a rural area. Just over half of the Cuban-trained participants (52%) reported that they planned to be certified in a specialty and 69% of the South African-trained participants planned to be certified in a specialty. Most Cuban-trained participants (90%) planned to practice in an underserved area regardless of location. Only a quarter of South African-trained participants planned to practice in an underserved area, and half of these planned to care primarily for an urban population. Cuban-trained participants (69%) planned to work inside their country of primary residence, whereas 45.2% of the South African-trained participants indicated that they were undecided.

Moreover, the internal consistency of items in each domain was high (Cronbach’s alpha ranged from 0.96 to 0.84). Independent sample *t*-tests of the scores on each domain showed that compared to South African-trained participants, Cuban-trained participants reported more favourable attitudes towards medical education subjects t(68) = 4.67, *p* < .001, higher confidence in health communication knowledge and skills *t*(69) = 3.27, *p* < .01, and higher confidence in performing clinical skills without direct supervision *t*(69) = 5.81, *p* < .001. Confidence in knowledge and skills acquired in clinical work between Cuban-trained participants and South African-trained participants was similar (*t*(69) = 1.18, *p* > .05). These findings are consistent with the detailed item-based analysis (see Table 6).

**Table 6:** Summary Findings of Educational Domains, comparing Cuban-trained and South African-trained Participants

Domain	α	*n*	Cuban-trained		South African-trained	95% CL	*t*	df
			Mean(*SD*)	*n*	Mean(*SD*)			
Appropriateness of training	.96	28	4.31(0.57)	42	3.51(0.78)	[0.46, 1.14]	4.67***	68
Knowledge and skills acquired in talking to patients, relatives of patients and other professional team members	.84	29	4.52(0.48)	42	4.12(0.53)	[0.16, 0.65]	3.27**	69
Knowledge and skills acquired in clinical work	.87	29	4.34(0.79)	42	4.14(0.61)	[−0.14, 0.53]	1.18	69
Current ability to perform clinical skills without direct supervision	.92	29	4.35(0.70)	42	3.30(0.78)	[0.69, 1.41]	5.81***	69

α = Cronbach’s alpha *p < .05 **p < .01 ***p < .001

### Qualitative findings

The main themes that emerged from interviews and focus group discussions were: a) the appropriateness of training in Cuba due to the lack of exposure to diseases common in South Africa (TB, HIV, trauma); b) the process of assimilation on return from Cuba; c) the range of skills acquired in Cuba; and d) career aspirations in South Africa.

### Is training in Cuba appropriate?

The burdens of disease in Cuba are quite different from those experienced in South Africa.

*“They just don’t get enough exposure. TB, HIV, trauma, you know, basic skills like delivering babies, basic surgery. They have to actually be a hands-on doctor that can do things like appendectomies and caesarean sections.” [Senior Faculty, SF]*

Competence in conducing surgical procedures and giving anaesthetics was a recurring theme among all participants. The issue of competencies in Caesarean section had dominated debates about the quality of Cuban-trained doctors and was acknowledged. More reasonable expectations of those trained in South African medical schools were mentioned.

*“The skills they acquire is being able to assess, to know which one is high risk pregnancy and when to refer and to know the warning signs for example like in obstetrics and then to know which patient will need to have a Caesarean section done.” [SF]*

*“So that [training in Cuba] means they do not come back with what we would expect as core knowledge of the diseases”[SF]*

Cuban-trained doctors felt that they have different core skills. They are more ‘hands-on’ in managing patients. Although they are not exposed to TB, HIV and trauma in the Cuban education system, the training emphasises flexibility and adaptability to all medical settings.

*“Cuban training allows us to be flexible in all settings… gives us hope to say something could be done to improve the health status as we move towards re engineering of primary health care Cuban South African-trained doctors become more relevant”[Cuban- trained doctor, CD]*

Some of the South African medical schools have developed more community orientated approaches which mirror some aspects of the Cuban approach.

*“They try to introduce us to the community and we work with patient. They start our training very early which is a good thing because, by the time we reach our final year we have better understanding working with patients not just like theory theory theory… So they try to make us interact with patients at a very, early, age which is a good thing.” (South African-trained student, SAS)*

The perception that the Cuban programme is not welcomed in most South African universities was strongly felt by returning participants.

*“There are constant comparisons between Cuban-trained doctors vs South African-trained doctors which creates stigma.” [Cuban- trained student, CS]*

Cuban-trained participants viewed their experience very favourably. They found the emphasis was on the community rather than solely on the patient. Community medicine had a dominant position compared to other courses and rotations. Teaching was also conducted at the community level and students visited patients at home.

*“Medicine is the same in the world, what makes Cuba different is the constant public health references where you needed to always go back to where the problem initiated. The disease was always linked to what was happening in the community, it made us realise that closing the tap was more important than mopping the floor.” [CS]*

The Cuban polyclinics allowed students from different disciplines (medicine, dentistry, physiotherapy etc.) to form teams to analyse the patient holistically. They viewed this is a vital part of their experience that would not be gained in South Africa.

Being trained in Spanish language was perceived as a considerable barrier by senior faculty. Some faculty felt that fluency in Afrikaans as well as English was necessary for some situations.

*“Their biggest weakness is that they have to translate…[from Spanish to English]”[SF]*

South African-trained students had a different view.

*“I don’t think it’s the language, I think actually the system of education. The teaching system in Cuba and the teaching system here I think it’s different … we do a clinical rotation in seven weeks so to adapt to a completely new system in seven weeks it’s quite a challenge”[SAS]*

However, South African-trained students also have language problems given the many different languages spoken by their patients and families.

“*At first it was really difficult because the languages are completely foreign to me, I didn’t understand it, we are able to compromise using English and okay using Zulu,… but eventually as you spend more time with them you end up learning the language.”[SAS]*

By contrast, Cuban-trained doctors claimed that spending a year learning Spanish (among other subjects) made them feel comfortable living in Cuba. South Africans were the fastest learners among foreign students in Cuba. Being exposed to a different language has made them more culturally adaptable to different settings and brings confidence when conversing with patients.

### The process of assimilation

Returning to South Africa was often a challenge, a source of anxiety and humiliation. Several Cuban-trained participants found it hard to understand why, after six years study in Cuba, some of them were “*busted down to 3*^*rd*^ *year medical students*”. There was concern that the duration of additional training required of the South African system appeared arbitrary with some returnees doing a year and others up to three years of study. Deans of medical schools were aware of these issues but felt that the only way to deal with their “*deficiencies*” was for them to re-do a lot of their medical training in South Africa. One had a different view:

*“Let them come back to South Africa as trained doctors, then put them in communities under supervised interns, and let them learn on the job. Don’t humiliate them by putting them through the examination system here because that leads to other problems. I think it makes them feel like lesser beings and it makes them feel like second class citizens, because they feel stupid having to pass the exam that they repeatedly fail very often.”[SF]*

Even before the return, students in Cuba were worried about what to expect.

*“The news we received from South Africa about the high number of failing students in South Africa were unforgettable. One knew that we were starting our final year on a negative mark. We had to work not double but triple to prove ourselves.”[CS]*

Returning graduates found strength in supporting each other and keeping in touch, exchanging notes on how to manage. Many of them wanted, and were expected, to return to their village of origin to set up health services for their local community. Some participants promoted Cuban education as providing the means of meeting the health challenges faced by South Africa. However, the South African-trained peers were critical about the way Cuban-trained students behaved and focused on the different patterns of disease and health system in Cuba and offered negative stereotypes.

*“They like to group themselves, as you know, we are Cubans, we will stick together we will speak our Spanish together. They don’t want to, you know, juggle with us whose studying medicine in South Africa because they don’t understand how things are done here you know, they will go and read their books in their rooms whereas the medicine here in Medunsa in your clinical years is in the hospital, it’s not in your text book.”[SAS]*

*“The medicine in Cuba is totally different from what we practice in South Africa so I don’t think it’s a good programme… I honestly don’t think it’s helping them in any how because everyone in South Africa, you go and ask… that hospital is infested with Cubans it’s so bad…”[SAS]*

### Skills acquired in Cuba

Despite these criticisms of their training some medical faculty acknowledged that Cuban-trained graduates and doctors had strengths in disease prevention, health promotion, acting as agents of change, and leadership skills.

*“Certainly, on the disease prevention, health promotion side and having empathy and so on, those are the things that we can learn from.”[SF]*

*“They are much more in tune with their role as an agent of change in society. They take on leadership roles to make things better in the system, not just the individual patient.”[SF]*

Other senior faculty said that their universities were already carrying out a lot of community-based teaching and had made many innovations in the curriculum.

*“I think all the approaches to the community we are already do so I do not think we would buy anything from the Cuban program.” [SF]*

Cuban-trained doctors affirmed these differences, noting that South African-trained doctors focus on the curative approach, viewing innovations in treatments as most important which creates tensions in relations with Cuban-trained doctors who are more focused on innovations for reengineering of Primary Health Care systems. They also noted the way the programme was not valued.

*“The whole program was used to fight political battles, not viewed as part of a solution in human resource improvement in our country. The government program was never internalized by our very own south African universities. Hence there is resistance in forming part of the solution”[CD]*

### Career aspirations

Deans of medical schools understood the primary health care and preventive orientation among Cuban-trained doctors but stated the importance of being able to cope with the curative needs of the population and viewed specialisation as an indicator of successful career building.

*“…these poor kids may find themselves isolated and not being able to proceed and progress and enrich their academic development in terms of specialising.”[SF]*

*“And what to me is much more rewarding and also makes me happy is the fact that they (black students) are now not only specialising here <this South African university>. They are also finding it within their capability to go and specialize in the other <South African> institutions.”[SF]*

There was a view that Cuban training was relevant to the needs of South Africa but it was a problem of where these returning doctors would fit.

*“It would be relevant I don’t want to lie it would be relevant that it has more of the preventive aspect to it, so therefore these guys… I would feel that they would be better suited for the national health insurance because national health insurance is mainly preventive medicine so you have a cadre of doctors who have already been schooled in that, most of their training.”[SF]*

By contrast, the returning students had a clear view of their career route and where they were most needed.

*“… basically I like to help the community because I’m from a rural community so where we have little health facilities so most of the people in the community they don’t have much medical knowledge - so yes, and I’ll be the first one in the community so, I would like to make a change.”[CS]*

South African-trained students, despite studying at community orientated medical school, viewed the prospect of working in a rural area as challenging because the lack of resources resulted in inadequate care.

*“I remember at one point there was this woman, like who was big but they didn’t have like correct sizes of cuff so like in times and in places like that you end up not treating patients well because of that limited resources”[SAS]*

Moreover, the aspirations of these students were more focused on specialisation and private practice career paths.

*“But I think the other problem lies, within us as health professionals you know. We learn skills through the poor because that is where we learn our medicine. But once we qualify we you know, we sort of distance ourselves from the people that we learn from, the people that gave us, you know medicine, the practice of medicine. Now people want to go into private, now people don’t want to work in the public sector…”[SAS]*

### Triangulation of findings

Both quantitative and qualitative findings highlighted the greater exposure to community experience and public health orientation of Cuban-trained participants. However, qualitative findings emphasized their limited exposure to diseases common in South Africa (TB, HIV) which fed into negative views of Cuban training and the program overall, particularly among South African-trained students. By contrast, quantitative findings on ability to perform clinical skills were stronger among Cuban- than South African-trained participants. Both quantitative and qualitative findings supported the abilities of Cuban-trained participants in having a role to play in serving rural and under-served urban areas and in community leadership.

## Discussion

We posed the question: how does Cuban medical training in terms of perceived quality of teaching, competencies achieved, and career plans compare with South African medical training? Cuban medical education is centred around public health and primary care, which are not as strongly endorsed by South African-trained graduates. The Cuban-trained graduates lacked exposure to diseases common in South Africa but rare in Cuba which has resulted in negative views of their skills. Their training does not equip them well for acute and curative aspects of hospital medicine, including obstetrics and anaesthetics, but their subsequent training in South Africa is intended to fill these skill gaps. The career aspirations expressed by Cuban participants reflect both their training in Cuba and their aspirations to serve rural and underserved communities from which they are drawn.

HTA approaches are typically used in evaluating new technologies from a range of perspectives, including safety, effectiveness, economic attributes, social, ethical and political impacts [23]. Innovations in medical education are not usually put through a HTA but in the Mandela-Castro programme there are concerns that cannot be easily resolved without such an assessment. We were not able to conduct a comprehensive HTA which would cover concerns about safety for students (e.g. stigma, mental health issues), effectiveness of the programme (e.g. number of graduates working in rural or under-served urban areas), economic issues (e.g. cost comparisons per doctor working in primary care), social and political issues (e.g. inequity in health care provision) and would provide a strong basis for making decisions about the future of the programme.

There are some major limitations to our study. First, it was not possible to obtain a random sample of medical students and graduates from South African medical schools. Second, our quantitative findings may reflect selection effects with participants with more favourable views taking part. Response biases (e.g. aquiescence bias, social desirability bias) may have influenced our findings. Third, our sample size is small and consequently it is not possible to examine sub-groups of participants to make more statistically powerful comparisons. Fourth, our field work was conducted during a time of extreme disruption in South African universities (many of them closed for long periods of time), rioting on campuses and high levels of suspicion among faculty and students. Finally, it would have been useful to conduct comparative studies in Cuba of South African and Cuban students and to make prospective assessments of Cuban-trained students in Cuba, on return to South African and following qualification. Such studies were beyond the scope of this project but would provide valuable information for medical health workforce planning for South Africa.

Despite these limitations in the research, we believe that our qualitative findings go some way to dispelling the overall negative narrative that has arisen around these Cuba-trained doctors. The media have reported a continuing rural health care crisis with a critical shortage of doctors [24]. While all doctors graduating from South African institutions are required to do placements in rural and under-served urban areas, long-term retention in rural areas is low. The current approach of re-badging returning Cuban students as South African doctors by putting them through 1 to 3 years of additional medical school study may undermine their commitment to work in rural areas post-graduation negating positive aspects of their Cuban experience. A more focused training programme based in rural clinics and peripheral hospitals could be explored.

### Previous research

A recent study from the University of Kwa-Zulu Natal explored the perceptions of teaching among 11 Cuban-trained graduates undertaking additional attachments [25]. They found that there were self-reported gaps in perceived ability in 35 of 75 skills and that students stated they could not perform 71 out of 75 skills. No comparisons with students trained in South Africa were made.

Cuban medical education initiatives have been studied in Portuguese speaking African countries and have concluded that investments in medical education have not been as efficient as hoped. More attention to the socialisation and role modelling processes at medical school might help and opportunities for postgraduate training in country might improve retention [26, 27].

### Policy issues

South Africa’s human resources for health strategy [28] emphasises reorientation towards public health and primary health care to meet the health needs of poor South Africans. It mentions Cuba’s significant improvement in health outcomes linked with training in public health. However, it does not acknowledge the importance of these Cuban-trained doctors in meeting South Africa’s human resource needs.

The Health Professions Council of South Africa are considering ‘absorbing’ Cuban-trained doctors into the hospital internships rather than innovative programmes of rural internships [29]. Hospital internships in sub-speciality medicine are unlikely to deliver improvements in primary health care. Extending medical internships to community facilities in rural and urban under-served areas would require a major policy shift, but given the potential benefits is worth considering.

Attempts to assimilate returning Cuban-trained students will require a reframing of the current negative narrative. Having a very large cadre of medically trained black South Africans returning who want to work in South Africa’s most difficult areas is timely. Their skills in primary care can be used to build infrastructure in rural and under-served areas. Acknowledgment that they are part of the solution to the health problems facing South Africa will be an essential step.

## Conclusion

Our study has several limitations that limit the conclusions that can be made. Our findings demonstrate that self-reported information about knowledge, skills and career aspirations can be obtained. Further national prospective comparative surveys of Cuban-trained and South African-trained students and doctors would provide valuable data for curriculum development and health workforce planning. There are indications that the process of assimilation on return for Cuban-trained students requires detailed evaluation to underpin improvements. However, it also appears clear that given the significant investments made in training South Africans in Cuba, attempts to assimilate returning Cuban-trained students will require a reframing of the current negative narrative, which need to focus on positive aspects of their training, orientation towards primary care and public health, and their aspirations to work in rural and under-served urban areas, if the increased value is to be obtained from that investment. Cuban-trained doctors are currently perceived as a problem but could, if seen as an asset, become part of the solution to South Africa’s health workforce problems.

## Additional files

Additional file 1: Medical Student Questionnaire. (DOCX 147 kb)

Additional file 2: Cuban Medical graduate Questionnaire. (DOCX 155 kb)

Additional file 3: Interview schedule. (DOCX 19 kb)

Additional file 4: Focus group schedule. (DOCX 21 kb)

Additional file 5: Descriptive data (numbers, percentages) for Likert scale scores. (DOCX 22 kb)

## Data Availability

The datasets used and/or analysed during the current study are available from the corresponding author on reasonable request.

## Abbreviations

CD: Cuban- trained doctor
CS: Cuban- trained student
HTA: Health Technology Assessment
SAS: South African-trained student
SF: Senior Faculty
