# Supplementary material for: Cuban medical training for South African students: a mixed methods study
Source: BMC Med Educ. 2019 Jun 17;19:216. doi: 10.1186/s12909-019-1661-4 (PMC6580452; doi:10.1186/s12909-019-1661-4)
Supplement: Supplementary file 1 — Medical Student Questionnaire. (DOCX 147 kb) [file 12909_2019_1661_MOESM1_ESM.docx]

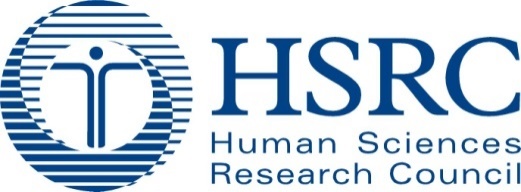


**SELF-COMPLETED QUESTIONNAIRE FOR MEDICAL HEALTH PROFESSIONALS**

Thank you for agreeing to complete this questionnaire.

The questions covers your career aspirations, planning and interests, your experience of medical education, your knowledge and skills acquired and your wellbeing. The survey will take approximately 20 minutes to complete.

The data in this form is collected in a way that does not allow individuals to be identified. Your participation in this survey is voluntary. Not taking part will have no adverse consequences for you. All your answers will be treated as confidential and will only be used by the research team.

1. **Personal details**

Please fill in the following details:

1. Your current status Tick box

Intern

Health professional

1. Year of graduation

(e.g. graduated in 2013, etc)

Your answer ________________________________

1. Please indicate the University in which you received the Cuban medical education training: __________________________________________________
2. Please indicate the University in which you received your South African orientation training on your return:

__________________________________________________

1. Nationality

South African Other

Please specify: ________________________

| 1. Race   Black African | Coloured | Asian/Indian | | White |
| --- | --- | --- | --- | --- |
| Other | Specify: _____________ | | | |
| 1. Male | Female | | | |
| 1. Married | Single | Divorced | Widow/Widower | |
| 1. Age | j.Number of dependents (not including a spouse/partner) | | | |
| 1. Father’s education | Primary school | Secondary school | | Technical |
|  | University | Not educated | | I don’t know |
| 1. Mother’s education | Primary School Secondary school Technical | | | |

University Not educated I don’t know

1. Your learning ability: Fast learner Average Slow learner

**2. Choice of medicine as a career**

When deciding to study medicine, how important were the following considerations? Use the scale from 1 (not important at all) to 5 (extremely important). Please circle the number.

| **Considerations** | **1**  **Not important at all** | **2**  **Of little importance** | **3**  **Moderately important** | **4**  **Important** | **5**  **Extremely**  **important** |
| --- | --- | --- | --- | --- | --- |
| 1. Family wanted me to be a doctor | 1 | 2 | 3 | 4 | 5 |
| 2. Good at sciences | 1 | 2 | 3 | 4 | 5 |
| 3. Working for social change | 1 | 2 | 3 | 4 | 5 |
| 4. High income potential | 1 | 2 | 3 | 4 | 5 |
| 5. Desire to work in a rural/underserved area | 1 | 2 | 3 | 4 | 5 |
| 6. Desire to work in another country | 1 | 2 | 3 | 4 | 5 |
| 7. Social recognition or status | 1 | 2 | 3 | 4 | 5 |
| 8. Stable, secure future | 1 | 2 | 3 | 4 | 5 |
| 9. Creativity and initiative | 1 | 2 | 3 | 4 | 5 |
| 10. Availability of jobs in the future | 1 | 2 | 3 | 4 | 5 |
| 11. Work/life balance | 1 | 2 | 3 | 4 | 5 |
| 12.Could not do my preferred subject/option | 1 | 2 | 3 | 4 | 5 |
| 13. Desire to help other people | 1 | 2 | 3 | 4 | 5 |
| 14. Improve health in my country | 1 | 2 | 3 | 4 | 5 |
| 15. Become a community leader | 1 | 2 | 3 | 4 | 5 |

**3. Choice of medical school**

When thinking about your choice of Cuban medical school, how important are the following considerations? Use the scale from 1 (not important at all) to 5 (extremely important). Please circle the number.

| **Considerations** | **1**  **Not important at all** | **2**  **Of little importance** | **3**  **Moderately important** | **4**  **Important** | **5**  **Extremely**  **important** |
| --- | --- | --- | --- | --- | --- |
| 1. Geographic location | 1 | 2 | 3 | 4 | 5 |
| 2. Reputation of the school | 1 | 2 | 3 | 4 | 5 |
| 3. Low cost/free course | 1 | 2 | 3 | 4 | 5 |
| 4. Availability of scholarships | 1 | 2 | 3 | 4 | 5 |
| 5. Meeting staff and/or students | 1 | 2 | 3 | 4 | 5 |
| 6. Quality of facilities | 1 | 2 | 3 | 4 | 5 |
| 7. Teaching methods | 1 | 2 | 3 | 4 | 5 |
| 8. Rural experience | 1 | 2 | 3 | 4 | 5 |
| 9. Family medicine experience | 1 | 2 | 3 | 4 | 5 |
| 10. Community/public health experience | 1 | 2 | 3 | 4 | 5 |
| 11.Opportunity for research experience/ MSc/PhD degree | 1 | 2 | 3 | 4 | 5 |
| 12. Sense of safety | 1 | 2 | 3 | 4 | 5 |
| 13. Influence of family/spouse/partner | 1 | 2 | 3 | 4 | 5 |
| 14. International opportunities | 1 | 2 | 3 | 4 | 5 |

**4. Career plans and interests**

When thinking about your career path when you were a Cuban medical student, how important were the following considerations? Use the scale from 1 (not important at all) to 5 (extremely important). Please circle the number.

| **Considerations** | **1**  **Not important at all** | **2**  **Of little importance** | **3**  **Moderately important** | **4**  **Important** | **5**  **Extremely**  **important** |
| --- | --- | --- | --- | --- | --- |
| 1. Working for social change | 1 | 2 | 3 | 4 | 5 |
| 2. High income potential | 1 | 2 | 3 | 4 | 5 |
| 3. Social recognition or status | 1 | 2 | 3 | 4 | 5 |
| 4. Stable, secure future | 1 | 2 | 3 | 4 | 5 |
| 5. Creativity and initiative | 1 | 2 | 3 | 4 | 5 |
| 6. Solidarity with disadvantaged people | 1 | 2 | 3 | 4 | 5 |
| 7. Availability of jobs | 1 | 2 | 3 | 4 | 5 |
| 8. Leadership potential | 1 | 2 | 3 | 4 | 5 |
| 9. Work/life balance | 1 | 2 | 3 | 4 | 5 |
| 10. Ability to pay off debt | 1 | 2 | 3 | 4 | 5 |
| 11. Opportunity for innovation | 1 | 2 | 3 | 4 | 5 |
| 12. Ability to make a difference | 1 | 2 | 3 | 4 | 5 |
| 13. Work with poor people | 1 | 2 | 3 | 4 | 5 |
| 14. Improve inequalities in society | 1 | 2 | 3 | 4 | 5 |

**5. Your experience of medical education**

a) How well did your study of the following basic medical sciences and other topics in Cuban medical school prepare you for clinical studies? Use the scale from 1 (very poor) to 5 (excellent). Please circle the number.

| **Topics** | **Not studied** | **1**  **Very poor** | **2**  **Not good** | **3**  **Fair** | **4**  **Good** | **5 Excellent** |
| --- | --- | --- | --- | --- | --- | --- |
| 1. Biochemistry |  | 1 | 2 | 3 | 4 | 5 |
| 2. Biostatistics and epidemiology |  | 1 | 2 | 3 | 4 | 5 |
| 3. Genetics |  | 1 | 2 | 3 | 4 | 5 |
| 4. Gross anatomy/dissection |  | 1 | 2 | 3 | 4 | 5 |
| 5. Immunology |  | 1 | 2 | 3 | 4 | 5 |
| 6. Introduction to clinical medicine |  | 1 | 2 | 3 | 4 | 5 |
| 7. Social science, ethics, politics |  | 1 | 2 | 3 | 4 | 5 |
| 8. Microanatomy/Histology |  | 1 | 2 | 3 | 4 | 5 |
| 9. Microbiology |  | 1 | 2 | 3 | 4 | 5 |
| 10. Neuroscience |  | 1 | 2 | 3 | 4 | 5 |
| 11. Pathology |  | 1 | 2 | 3 | 4 | 5 |
| 12. Pharmacology |  | 1 | 2 | 3 | 4 | 5 |
| 13. Physiology |  | 1 | 2 | 3 | 4 | 5 |
| 14. Psychology |  | 1 | 2 | 3 | 4 | 5 |
| 15. Pathophysiology of disease |  | 1 | 2 | 3 | 4 | 5 |
| 16. Introduction to the patient |  | 1 | 2 | 3 | 4 | 5 |
| 17. Research ethics |  | 1 | 2 | 3 | 4 | 5 |
| 18. Foreign languages |  | 1 | 2 | 3 | 4 | 5 |

b) What was the quality of the facilities at your Cuban medical school? Use the scale from 1 (very poor) to 5 (excellent). Please circle the number.

| **Facilities** | **Not available** | **1**  **Very poor** | **2**  **Not good** | **3**  **Fair** | **4**  **Good** | **5**  **Excellent** |
| --- | --- | --- | --- | --- | --- | --- |
| 1. Anatomy dissection laboratory |  | 1 | 2 | 3 | 4 | 5 |
| 2. Physiology/biochemistry laboratories |  | 1 | 2 | 3 | 4 | 5 |
| 3. Microscopes, histology collections |  | 1 | 2 | 3 | 4 | 5 |
| 4. Library |  | 1 | 2 | 3 | 4 | 5 |
| 5. Computer laboratory |  | 1 | 2 | 3 | 4 | 5 |
| 6. E-learning |  | 1 | 2 | 3 | 4 | 5 |
| 7. Actor patients: communication |  | 1 | 2 | 3 | 4 | 5 |
| 8. Lecture theatres: size, acoustics, lighting, projection |  | 1 | 2 | 3 | 4 | 5 |
| 9. Small group teaching rooms |  | 1 | 2 | 3 | 4 | 5 |
| 10. Teaching rooms in hospitals |  | 1 | 2 | 3 | 4 | 5 |
| 11. Teaching rooms in clinics, community |  | 1 | 2 | 3 | 4 | 5 |
| 12. Language laboratory |  | 1 | 2 | 3 | 4 | 5 |
| 13. Access to computers for own use |  | 1 | 2 | 3 | 4 | 5 |

c) How well do you think areas of learning were covered in your Cuban medical school? Use the scale from 1 (very poor) to 5 (excellent). Please circle the number.

| **Areas** | **Not covered** | **1**  **Very poor** | **2**  **Not good** | **3**  **Fair** | **4**  **Good** | **5**  **Excellent** |
| --- | --- | --- | --- | --- | --- | --- |
| 1. Diagnosis of disease |  | 1 | 2 | 3 | 4 | 5 |
| 2. Management of disease |  | 1 | 2 | 3 | 4 | 5 |
| 3. Health maintenance |  | 1 | 2 | 3 | 4 | 5 |
| 4. Public health |  | 1 | 2 | 3 | 4 | 5 |
| 5. Role of community health and social service agencies |  | 1 | 2 | 3 | 4 | 5 |
| 6. Disease prevention |  | 1 | 2 | 3 | 4 | 5 |
| 7. Women's health |  | 1 | 2 | 3 | 4 | 5 |
| 8. Child health |  | 1 | 2 | 3 | 4 | 5 |
| 9. Culturally appropriate care for diverse populations |  | 1 | 2 | 3 | 4 | 5 |
| 10. Social determinants of health |  | 1 | 2 | 3 | 4 | 5 |
| 11. Politics of health |  | 1 | 2 | 3 | 4 | 5 |
| 12. Health economics |  | 1 | 2 | 3 | 4 | 5 |
| 13. Evidence based health care |  | 1 | 2 | 3 | 4 | 5 |
| 14. Team management skills |  | 1 | 2 | 3 | 4 | 5 |
| 15. Leadership skills |  | 1 | 2 | 3 | 4 | 5 |
| 16. Taking responsibility for your actions |  | 1 | 2 | 3 | 4 | 5 |

d) Do you agree or disagree with these statements about examinations at your Cuban medical school? Use the scale from 1 (strongly disagree) to 5 (strongly agree). Please circle the number.

| **Examinations** | **1**  **Strongly disagree** | **2**  **Disagree** | **3**  **Neutral** | **4**  **Agree** | **5**  **Strongly agree** |
| --- | --- | --- | --- | --- | --- |
| 1. Pre-clinical exams tested what I had been taught fairly | 1 | 2 | 3 | 4 | 5 |
| 2. Pre-clinical exams were too hard | 1 | 2 | 3 | 4 | 5 |
| 3. Clinical exams tested my skills fairly | 1 | 2 | 3 | 4 | 5 |
| 4. Clinical exams were too hard | 1 | 2 | 3 | 4 | 5 |
| 5. There were too many exams | 1 | 2 | 3 | 4 | 5 |
| 6. I performed well in exams | 1 | 2 | 3 | 4 | 5 |

e) Consider how much of the following items were an indication of academic stress for you at your Cuban medical school. Use the scale from 1 (not at all stressful) to 5 (extremely stressful). Please circle the number.

| **Stressors** | **1**  **Not at all stressful** | **2**  **Slightly stressful** | **3**  **Stressful** | **4**  **Very stressful** | **5**  **Extremely stressful** |
| --- | --- | --- | --- | --- | --- |
| 1. Formal academic exams | 1 | 2 | 3 | 4 | 5 |
| 1. Other assessments | 1 | 2 | 3 | 4 | 5 |
| 1. Regular study assignments | 1 | 2 | 3 | 4 | 5 |
| 1. Academic atmosphere | 1 | 2 | 3 | 4 | 5 |
| 1. Physical and social conditions | 1 | 2 | 3 | 4 | 5 |
| 1. Study demands and leisure activities | 1 | 2 | 3 | 4 | 5 |
| 1. The amount of material to study | 1 | 2 | 3 | 4 | 5 |
| 1. Keeping up with studies | 1 | 2 | 3 | 4 | 5 |
| 1. Faculty-student relations | 1 | 2 | 3 | 4 | 5 |
| 1. Time pressure | 1 | 2 | 3 | 4 | 5 |
| 1. Presenting cases/reports before class/group | 1 | 2 | 3 | 4 | 5 |

f) In your Cuban medical education experience, have you participated in any required curricular activities where you had the opportunity to learn with students from different health professions? Yes No

If yes, please specify which professions: ______________________________________

g) What are your views about learning with other health professionals? Use the scale from 1 (strongly disagree) to 5 (strongly agree). Please circle the number.

|  | **1**  **Strongly disagree** | **2**  **Disagree** | **3**  **Neutral** | **4**  **Agree** | **5**  **Strongly agree** |
| --- | --- | --- | --- | --- | --- |
| The learning experience with other health professions students helped me gain a better understanding of the roles of other professions in care of patients. | 1 | 2 | 3 | 4 | 5 |

**6. Knowledge and skills acquired in talking to patients, relatives of patients and other professional team members**

Please rate your confidence in these areas from not at all confident to very confident. Use the scale from 1 (not all confident) to 5 (very confident). Please circle the number.

| **Communication skills** | **1**  **Not at all confident** | **2**  **Not confident** | **3**  **Unsure** | **4**  **Confident** | **5**  **Very confident** |
| --- | --- | --- | --- | --- | --- |
| 1. Elicit patients’ questions, their understanding of their condition and treatment options, and their views, concerns, values and preferences | 1 | 2 | 3 | 4 | 5 |
| 2. Communicate clearly, sensitively and empathically with patients, relatives or other careers | 1 | 2 | 3 | 4 | 5 |
| 3. Communicate appropriately in difficult circumstances (e.g. with difficult or violent patients, when breaking bad news, or with vulnerable patients) | 1 | 2 | 3 | 4 | 5 |
| 4. Communicate health plans with local communities | 1 | 2 | 3 | 4 | 5 |
| 5. Know when to seek help from a senior colleague | 1 | 2 | 3 | 4 | 5 |
| 6. Learn and work effectively within a multi-professional team | 1 | 2 | 3 | 4 | 5 |

**7. Knowledge and skills acquired in clinical skills**

a) Please rate your confidence in these areas from not at all confident to very confident. Use the scale from 1 (not all confident) to 5 (very confident). Please circle the number.

| **Clinical skills** | **1**  **Not at all confident** | **2**  **Not confident** | **3**  **Unsure** | **4**  **Confident** | **5**  **Very confident** |
| --- | --- | --- | --- | --- | --- |
| 1. Provide cardio-pulmonary resuscitation | 1 | 2 | 3 | 4 | 5 |
| 2. Carry out practical procedures: venepuncture, taking blood cultures, measuring blood glucose | 1 | 2 | 3 | 4 | 5 |
| 3. Establish peripheral intravenous access (set up an IV drip) | 1 | 2 | 3 | 4 | 5 |
| 4. Carry out practical procedures: urinary catheterisation, skin suturing | 1 | 2 | 3 | 4 | 5 |
| 5. Prescribe, set up and monitor a blood transfusion | 1 | 2 | 3 | 4 | 5 |
| 6. Prescribe dose and route of insulin, including use of sliding scales | 1 | 2 | 3 | 4 | 5 |

b) How confident you are in your current ability to perform the following skills without direct supervision (i.e., with no supervisor in the room)? Use the scale from 1 (not all confident) to 5 (very confident). Please circle the number.

| **Skills** | **1**  **Not at all confident** | **2**  **Not confident** | **3**  **Unsure** | **4**  **Confident** | **5**  **Very confident** |
| --- | --- | --- | --- | --- | --- |
| 1. Diagnose and manage acute medical emergencies | 1 | 2 | 3 | 4 | 5 |
| 2. Obstetrics: carry out a forceps delivery | 1 | 2 | 3 | 4 | 5 |
| 3. Obstetrics: carry out a Caesarean section | 1 | 2 | 3 | 4 | 5 |
| 4. Give an anaesthetic for minor surgery | 1 | 2 | 3 | 4 | 5 |
| 5. Intubate and insert an endotracheal tube | 1 | 2 | 3 | 4 | 5 |
| 6. Give health promotion advice to mothers | 1 | 2 | 3 | 4 | 5 |
| 7. Conduct a health survey in a local community | 1 | 2 | 3 | 4 | 5 |
| 8. Manage a primary health care team | 1 | 2 | 3 | 4 | 5 |

**8. Ethnic identity**

a) These questions are about your ethnicity or your ethnic group and how you feel about it or react to it. Use the scale from 1 (strongly agree) to 5 (strongly disagree). Please circle the number.

| **Ethnic identity** | **1**  **Strongly agree** | **2**  **Agree** | **3**  **Unsure** | **4**  **Disagree** | **5**  **Strongly disagree** |
| --- | --- | --- | --- | --- | --- |
| 1. I have spent time trying to find out more about my ethnic group, such as its history, traditions, and customs. | 1 | 2 | 3 | 4 | 5 |
| 1. I am active in organizations or social groups that include mostly members of my own ethnic group. | 1 | 2 | 3 | 4 | 5 |
| 1. I have a clear sense of my ethnic background and what it means for me. | 1 | 2 | 3 | 4 | 5 |
| 1. I think a lot about how my life will be affected by my ethnic group membership. | 1 | 2 | 3 | 4 | 5 |
| 1. I am happy that I am a member of the group I belong to. | 1 | 2 | 3 | 4 | 5 |
| 1. I have a strong sense of belonging to my own ethnic group. | 1 | 2 | 3 | 4 | 5 |
| 1. I understand pretty well what my ethnic group membership means to me. | 1 | 2 | 3 | 4 | 5 |
| 1. In order to learn more about my ethnic background, I have often talked to other people about my ethnic group. | 1 | 2 | 3 | 4 | 5 |
| 1. I have a lot of pride in my ethnic group. | 1 | 2 | 3 | 4 | 5 |
| 1. I participate in cultural practices of my own group, such as special food, music, or customs. | 1 | 2 | 3 | 4 | 5 |
| 1. I feel a strong attachment towards my own ethnic group. | 1 | 2 | 3 | 4 | 5 |
| 1. I feel good about my cultural or ethnic background. | 1 | 2 | 3 | 4 | 5 |

**9. Career Planning**

| a. Are you planning to work in primary health care?   \| Yes \| No \| Undecided \| \| --- \| --- \| --- \|   b. Are you planning to become certified in a specialty (other than primary health care)? | | | | |
| --- | --- | --- | --- | --- | --- | --- | --- |
|  | Yes | No | | Undecided |
| If yes, which speciality? ______________________________ | | | | |
| c. How satisfied were you with your opportunities during medical school to explore potential career choices? | | | | |
| Very satisfied | Satisfied | Neutral | Dissatisfied Very dissatisfied | |
|  |  |  |  | |
| d. If you could revisit your career choice, would you still choose to attend medical school? | | | | |
| No | Probably not | Probably yes | | Yes |
| e. How much do you expect to be involved in research during your medical career? | | | | |
| Very much | Somewhat | Limited | | Not at all |
| f. Do you plan to practice in an underserved area? | | | | |
|  | Yes | No | | Undecided |
| g. What is the likely location? | | | | |
|  | Rural | Inner-city | | Other |
| h. Regardless of location, do you plan to care primarily for an underserved population? | | | | |
|  | Yes | No | | Undecided |
| i. Do you plan to work outside of your country of primary residence? | | | | |
| Yes | No | Undecided | | Not possible |
|  |  |  | |  |
| j. How much has your experience at medical school influenced these choices? | | | | |
| Very much | Somewhat | Limited | | Not at all |

**10. Please write here any other points about your medical education that you would like to make:**

**- THANK YOU FOR YOUR PARTICIPATION!** ☺ **-**
