# Supplementary material for: Cuban medical training for South African students: a mixed methods study
Source: BMC Med Educ. 2019 Jun 17;19:216. doi: 10.1186/s12909-019-1661-4 (PMC6580452; doi:10.1186/s12909-019-1661-4)
Supplement: Supplementary file 3 — Interview schedule. (DOCX 19 kb) [file 12909_2019_1661_MOESM3_ESM.docx]

**Semi-structured interview format for Cuban and African medical schools**

• Deans of medical schools

• Senior doctors and nurses from teaching hospitals/community and public services

**Interviewee details**

Date of interview Interviewer

Mode: Face-to-face/Skype/Telephone

Name Sex M/F

Institution

Country

Start time: Finish time:

Permission to record interview: YES/NO

**Introduction.**

Thank you for agreeing to be interviewed for this project. The Department of International Development, UK have funded us to get information to examine the role of the Cuban system of medical education in sub-Saharan Africa and how it compares with the medical education provided by existing schools.

Our interview will cover how your school works, what your school does, and what differences you make.

The interview will take about an hour.

**1. First, I’d like to ask you about how your school works**.

a) What do you believe in? (Prompt: What sort of shared values do people hold?)

b) Who do you serve? (Prompt: Do you have a defined catchment population?)

c) How do you work with others? (Prompt: What sort of partnerships do you have?)

d) How do you make decisions? (Prompt: Who is involved?)

**2. Second, I will turn to what your school does.**

a) Where do you invest resources? (Prompt: Do you attract additional funds; How do you allocate resources within the school?)

b) What, where and how do you teach? (Prompt: Would you say your school is traditional? Does the curriculum reflect local needs? What skills do you expect your graduates to have in obstetrics?)

c) Who do you teach? (Prompt: Do you have student recruitment policies? Are your students from poorer backgrounds or marginal ethnic groups? Do you recruit on academic ability mainly? Or do other factors play a role?)

d) Who does the teaching? (Prompt: Do you involve local community-based teachers?)

e) How does your research relate to your mission and values? (Prompt: Does research focus on local community issues?)

f) What contribution do you make to the delivery of health care? (Prompt: Does the way you train your students influence their career choices? Do your graduates work in rural and other disadvantaged communities?)

**3. Third, a big question - what difference do you make?**

a) What are your graduates doing? (Prompt: Do you have a system to follow up your graduates? Are they working in areas where they are most needed?)

b) What difference have you made to health and health services for your local community? (Prompt: Have health services improved or become more closely linked to local problems?)

c) How have you shared your ideas and influenced others? (Prompt: Do you have regular meetings with local policy makers?)

d) What impact have you made with other schools? (Prompt: Have you shared your curriculum or teaching materials with other schools?)

**FOR AFRICAN MEDICAL SCHOOL INTERVIEWEES ONLY**

**4. Turning to your experience of Cuban medical education,**

a) What are the differences between Cuban-trained and African-trained doctors? (Prompt: Are their skills similar? Are they motivated towards doing medicine in the same ways?)

b) Have you worked with any students or graduates from Cuban medical schools? (Prompt: What are their strengths and weaknesses?)

c) How does Cuban medical education fit with the health needs of your country? (Prompt: Do you think the Cuban curriculum is relevant to Africa? Do the graduates have the skills they need to practice effectively?)

d) Are there any things that you would like to take from the Cuban approach in your own school? (Prompt: What innovations would you like to see in your school?)

e) Does your school provide any courses for graduates from Cuban medical schools? (Prompt: What topics do you provide or would you like to provide?)

**FOR CUBAN MEDICAL SCHOOL INTERVIEWEES ONLY**

**4. Turning to your experience of African medical education,**

a) What are the differences between Cuban-trained and African-trained doctors? (Prompt: Are their skills similar? Are they motivated towards doing medicine in the same ways? )

b) Have you worked with students or graduates from Africa? (Prompt: What are their strengths and weaknesses?)

c) How does African medical education fit with the health needs of your country? (Prompt: Do you think the Cuban curriculum is relevant to Africa? Do the graduates have the skills they need to practice effectively?)

d) Are there any things that you would like to take from the African approach in your own school? (Prompt: What innovations would you like to see in your school?)

e) Does your school provide any courses for graduates from African medical schools? (Prompt: What topics do you provide or would you like to provide?)

**5. Finally, are there any other things about your school that make it special?**

I will send you a transcript of our interview which you can edit or add to as you wish.

Thank you for your involvement in our project. Your views will make an important contribution.
