# Supplementary material for: Cuban medical training for South African students: a mixed methods study
Source: BMC Med Educ. 2019 Jun 17;19:216. doi: 10.1186/s12909-019-1661-4 (PMC6580452; doi:10.1186/s12909-019-1661-4)
Supplement: Supplementary file 4 — Focus group schedule. (DOCX 21 kb) [file 12909_2019_1661_MOESM4_ESM.docx]

**Focus group discussion format**

**Group details**

Date of group discussion: Group leader:

Group: Pre-clinical/Clinical/Junior doctors Number participants:

Cuban/African/Mixed

Institution:

Country:

Start time: Finish time:

Permission to record discussion: YES/NO

**Introduction.**

Thank you for agreeing to join this discussion group. The Department of International Development, UK have funded us to get information to examine the role of the Cuban system of medical education in sub-Saharan Africa and how it compares with the medical education provided by other medical schools in the region.

Our discussion will cover how your reasons for doing medicine, your experience at medical school, your career options and the difference you are able to make.

The discussion will take about an hour.

**1. First, let’s discuss our values and reasons for doing medicine.**

a) Why did you choose to study medicine? (Prompt: What sort of motivation do you have?)

b) Who are you being trained to serve? (Prompt: What are the problems of working in disadvantaged communities?)

c) How should doctors work with other health professionals? (Prompt: Should the doctor be the leader? Do you know anything about the training of non-medical health professionals? Do you work mostly in a team or alone?)

d) Do you get to make decisions about your training or career? (Prompt: Do you get any advice or counselling about your career?)

**2. Now, let’s move to a second topic - your medical education and training.**

a) Why do you think you were selected by your school to do medicine? (Prompt: Just academic ability or were there other factors? Do you think the selection processes are fair? Did you have a choice of places to study medicine?)

b) How would you describe the methods of teaching at your school? (Prompt: Would you say your school is traditional? Does the curriculum reflect local needs? How much time do you spend in the local community health centres? Are there topics taught that seem to be of little value to you?)

c) What practical clinical skills do you expect to have once you have graduated? (Prompt: Will you be able to carry out operations like a Caesarean section? Or give an anaesthetic?)

d) Who does the teaching? (Prompt: Do local community-based doctors and other health professionals teach you?)

e) What contribution do you make to the delivery of health care? (Prompt: Do work in rural or other disadvantaged communities?)f) Did you experience any problems at medical school relating to your life as a student? (Prompt: assimilation, language, learning, loneliness)

**3. Third, a big question - what difference do you make?**

a) What are your plans for the future? (Prompt: Where do you expect to be working in 5-10 years time? Do you have a choice?)

b) What difference have you made to health and health services for your local community? (Prompt: Have you got the right skills to help patients? Do you need further training? Is so, what areas?)

c) Has your training influenced your career choice or where you want to work? (Prompt: What are the main factors that influenced you?

**FOR AFRICAN MEDICAL SCHOOL GROUPS ONLY**

**4. Finally, let’s talk about your experience of Cuban medical education,**

a) What are the differences between Cuban-trained and African-trained doctors? (Prompt: Are their skills similar? Are they motivated towards doing medicine in the same ways?)

b) Have you worked with any students or graduates from Cuban medical schools? (Prompt: What are their strengths and weaknesses?)

c) How does Cuban medical education fit with the health needs of your country? (Prompt: Do you think the Cuban curriculum is relevant to Africa? Do the graduates have the skills they need to practice effectively?)

d) Are there any topics/experiences that you think are so valuable that everyone studying medicine should have them? (Prompt: What about working in villages? Or with disadvantaged people?)

e) Are you getting any special courses to help you with your training? (Prompt: For example, languages, culture, and practical skills?)

**FOR CUBAN MEDICAL SCHOOL GROUPS ONLY**

**4. Finally, let’s talk about your experience of African medical education**

a) What are the differences between Cuban-trained and African-trained doctors? (Prompt: Are their skills similar? Are they motivated towards doing medicine in the same ways? )

b) Have you worked with students or graduates from Africa? (Prompt: What are their strengths and weaknesses?)

c) How does African medical education fit with the health needs of your country? (Prompt: Do you think the Cuban curriculum is relevant to Africa? Do the graduates have the skills they need to practice effectively?)

d) Are there any topics/experiences that you think are so valuable that everyone studying medicine should have them? (Prompt: What about working in villages? Or with disadvantaged people?)

e) Are you getting any special courses to help you with your training? (Prompt: For example, languages, culture, and practical skills?)

Thank you for your involvement in our project. Your views will make an important contribution.
