# Supplementary material for: Cuban medical training for South African students: a mixed methods study
Source: BMC Med Educ. 2019 Jun 17;19:216. doi: 10.1186/s12909-019-1661-4 (PMC6580452; doi:10.1186/s12909-019-1661-4)
Supplement: Supplementary file 5 — Descriptive data (numbers, percentages) for Likert scale scores. (DOCX 22 kb) [file 12909_2019_1661_MOESM5_ESM.docx]

**Descriptive data (numbers, Percentages) of responses to Likert scale questions**

Table 1. Experience of Basic Medical Science Topics

|  |  | Very poor | | Not good | | Fair | | Good | | Excellent | |
| --- | --- | --- | --- | --- | --- | --- | --- | --- | --- | --- | --- |
|  |  | n | % | n | % | n | % | n | % | n | % |
| Cuban-trained | [1. Biochemistry] | 0 | 0 | 0 | 0 | 3 | 10.7 | 13 | 46.4 | 12 | 42.9 |
|  | [2. Biostatistics and epidemiology] | 0 | 0 | 0 | 0 | 2 | 7.1 | 9 | 32.1 | 17 | 60.7 |
|  | [3. Genetics] | 0 | 0 | 0 | 0 | 10 | 35.7 | 7 | 25 | 11 | 39.3 |
|  | [4. Gross anatomy/dissection] | 1 | 3.6 | 0 | 0 | 4 | 14.3 | 7 | 25 | 16 | 57.1 |
|  | [5. Immunology] | 0 | 0 | 2 | 7.1 | 5 | 17.9 | 7 | 25 | 14 | 50 |
|  | [6. Introduction to clinical medicine] | 0 | 0 | 0 | 0 | 3 | 11.1 | 10 | 37 | 14 | 51.9 |
|  | [7. Social science, ethics, politics] | 0 | 0 | 2 | 7.4 | 6 | 22.2 | 6 | 22.2 | 13 | 48.1 |
|  | [8. Microanatomy/Histology] | 0 | 0 | 1 | 3.6 | 4 | 14.3 | 8 | 28.6 | 15 | 53.6 |
|  | [9. Microbiology] | 0 | 0 | 0 | 0 | 4 | 14.3 | 10 | 35.7 | 14 | 50 |
|  | [10. Neuroscience] | 0 | 0 | 1 | 3.7 | 11 | 40.7 | 8 | 29.6 | 7 | 25.9 |
|  | [11. Pathology] | 0 | 0 | 0 | 0 | 5 | 17.9 | 9 | 32.1 | 14 | 50 |
|  | [12. Pharmacology] | 0 | 0 | 0 | 0 | 4 | 14.3 | 12 | 42.9 | 12 | 42.9 |
|  | [13. Physiology] | 0 | 0 | 0 | 0 | 4 | 14.3 | 9 | 32.1 | 15 | 53.6 |
|  | [14. Psychology] | 0 | 0 | 0 | 0 | 1 | 3.6 | 11 | 39.3 | 16 | 57.1 |
|  | [15. Pathophysiology of disease] | 0 | 0 | 0 | 0 | 3 | 10.7 | 11 | 39.3 | 14 | 50 |
|  | [16. Introduction to the patient] | 0 | 0 | 0 | 0 | 2 | 7.1 | 5 | 17.9 | 21 | 75 |
|  | [17. Research ethics] | 0 | 0 | 1 | 3.6 | 3 | 10.7 | 11 | 39.3 | 13 | 46.4 |
|  | [18. Foreign languages] | 0 | 0 | 1 | 3.6 | 4 | 14.3 | 7 | 25 | 16 | 57.1 |
|  |  |  |  |  |  |  |  |  |  |  |  |

Table 1 (continued)

| South African-trained | [1. Biochemistry] | 1 | 2.4 | 5 | 11.9 | 18 | 42.9 | 14 | 33.3 | 4 | 9.5 |
| --- | --- | --- | --- | --- | --- | --- | --- | --- | --- | --- | --- |
|  | [2. Biostatistics and epidemiology] | 5 | 12.2 | 4 | 9.8 | 17 | 41.5 | 12 | 29.3 | 3 | 7.3 |
|  | [3. Genetics] | 3 | 7.1 | 3 | 7.1 | 19 | 45.2 | 14 | 33.3 | 3 | 7.1 |
|  | [4. Gross anatomy/dissection] | 2 | 4.8 | 3 | 7.1 | 9 | 21.4 | 14 | 33.3 | 14 | 33.3 |
|  | [5. Immunology] | 1 | 2.4 | 4 | 9.5 | 11 | 26.2 | 15 | 35.7 | 11 | 26.2 |
|  | [6. Introduction to clinical medicine] | 0 | 0 | 3 | 7.3 | 4 | 9.8 | 17 | 41.5 | 17 | 41.5 |
|  | [7. Social science, ethics, politics] | 7 | 16.7 | 3 | 7.1 | 19 | 45.2 | 6 | 14.3 | 7 | 16.7 |
|  | [8. Microanatomy/Histology] | 6 | 14.3 | 8 | 19 | 13 | 31 | 8 | 19 | 7 | 16.7 |
|  | [9. Microbiology] | 2 | 4.8 | 5 | 11.9 | 8 | 19 | 19 | 45.2 | 8 | 19 |
|  | [10. Neuroscience] | 2 | 4.9 | 6 | 14.6 | 12 | 29.3 | 12 | 29.3 | 9 | 22 |
|  | [11. Pathology] | 1 | 2.4 | 4 | 9.5 | 7 | 16.7 | 18 | 42.9 | 12 | 28.6 |
|  | [12. Pharmacology] | 3 | 7.1 | 7 | 16.7 | 9 | 21.4 | 9 | 21.4 | 14 | 33.3 |
|  | [13. Physiology] | 2 | 4.8 | 2 | 4.8 | 7 | 16.7 | 18 | 42.9 | 13 | 31 |
|  | [14. Psychology] | 5 | 11.9 | 6 | 14.3 | 14 | 33.3 | 9 | 21.4 | 8 | 19 |
|  | [15. Pathophysiology of disease] | 0 | 0 | 4 | 9.5 | 6 | 14.3 | 17 | 40.5 | 15 | 35.7 |
|  | [16. Introduction to the patient] | 1 | 2.4 | 3 | 7.3 | 5 | 12.2 | 14 | 34.1 | 18 | 43.9 |
|  | [17. Research ethics] | 9 | 22 | 5 | 12.2 | 14 | 34.1 | 8 | 19.5 | 5 | 12.2 |
|  | [18. Foreign languages] | 9 | 36 | 5 | 20 | 5 | 20 | 4 | 16 | 2 | 8 |

Table 2. Knowledge and Skills Acquired in Talking to Patients, Relatives of Patients and other Professional Team Members

|  |  | Not at all confident | | Not confident | | Unsure | | Confident | | Very confident | |
| --- | --- | --- | --- | --- | --- | --- | --- | --- | --- | --- | --- |
|  |  |  | % |  | % |  | % |  | % |  | % |
| Cuban-trained | [1. Elicit patients’ questions, their understanding of their condition and treatment options, and their views, concerns, values and preferences] | 0 | 0 | 0 | 0 | 0 | 0 | 12 | 41.4 | 17 | 58.6 |
|  | [2. Communicate clearly, sensitively and empathically with patients, relatives or other carers] | 0 | 0 | 0 | 0 | 1 | 3.4 | 10 | 34.5 | 18 | 62.1 |
|  | [3. Communicate appropriately in difficult circumstances (e.g. with difficult or violent patients, when breaking bad news, or with vulnerable patients)]* | 0 | 0 | 0 | 0 | 1 | 3.4 | 15 | 51.7 | 13 | 44.8 |
|  | [4. Communicate health plans with local communities] | 0 | 0 | 1 | 3.4 | 0 | 0 | 13 | 44.8 | 15 | 51.7 |
|  | [5. Know when to seek help from a senior colleague] | 0 | 0 | 0 | 0 | 0 | 0 | 13 | 44.8 | 16 | 55.2 |
|  | [6. Learn and work effectively within a multi-professional team] | 0 | 0 | 0 | 0 | 0 | 0 | 13 | 44.8 | 16 | 55.2 |
| South African-trained | [1. Elicit patients’ questions, their understanding of their condition and treatment options, and their views, concerns, values and preferences] | 0 | 0 | 0 | 0 | 3 | 7.1 | 22 | 52.4 | 17 | 40.5 |
|  | [2. Communicate clearly, sensitively and empathically with patients, relatives or other carers] | 0 | 0 | 1 | 2.4 | 3 | 7.1 | 17 | 40.5 | 21 | 50 |
|  | [3. Communicate appropriately in difficult circumstances (e.g. with difficult or violent patients, when breaking bad news, or with vulnerable patients)]* | 1 | 2.4 | 6 | 14.3 | 9 | 21.4 | 19 | 45.2 | 7 | 16.7 |
|  | [4. Communicate health plans with local communities] | 1 | 2.4 | 2 | 4.8 | 14 | 33.3 | 17 | 40.5 | 8 | 19 |
|  | [5. Know when to seek help from a senior colleague] | 0 | 0 | 0 | 0 | 1 | 2.4 | 18 | 42.9 | 23 | 54.8 |
|  | [6. Learn and work effectively within a multi-professional team] | 0 | 0 | 1 | 2.4 | 3 | 7.1 | 25 | 59.5 | 13 | 31 |

Table 3. Knowledge and Skills Acquired in Clinical Work

|  |  | Not at all confident | | Not confident | | Unsure | | Confident | | Very confident | |
| --- | --- | --- | --- | --- | --- | --- | --- | --- | --- | --- | --- |
|  |  | n | % | n | % | n | % | n | % | n | % |
| Cuban-trained | [1. Provide cardio-pulmonary resuscitation ] | 0 | 0 | 2 | 6.9 | 3 | 10.3 | 13 | 44.8 | 11 | 37.9 |
|  | [2. Carry out practical procedures: venepuncture, taking blood cultures, measuring blood glucose] | 0 | 0 | 1 | 3.4 | 1 | 3.4 | 8 | 27.6 | 19 | 65.5 |
|  | [3. Establish peripheral intravenous access (set up an IV drip)] | 0 | 0 | 2 | 6.9 | 0 | 0 | 8 | 27.6 | 19 | 65.5 |
|  | [4. Carry out practical procedures: urinary catheterisation, skin suturing] | 0 | 0 | 1 | 3.4 | 3 | 10.3 | 7 | 24.1 | 18 | 62.1 |
|  | [5. Prescribe, set up and monitor a blood transfusion] | 0 | 0 | 3 | 10.3 | 5 | 17.2 | 6 | 20.7 | 15 | 51.7 |
|  | [6. Prescribe dose and route of insulin, including use of sliding scales] | 0 | 0 | 2 | 6.9 | 5 | 17.2 | 6 | 20.7 | 16 | 55.2 |
| South African-trained | [1. Provide cardio-pulmonary resuscitation ] | 0 | 0 | 1 | 2.4 | 8 | 19 | 27 | 64.3 | 6 | 14.3 |
|  | [2. Carry out practical procedures: venepuncture, taking blood cultures, measuring blood glucose] | 0 | 0 | 0 | 0 | 0 | 0 | 5 | 11.9 | 37 | 88.1 |
|  | [3. Establish peripheral intravenous access (set up an IV drip)] | 0 | 0 | 1 | 2.4 | 1 | 2.4 | 11 | 26.2 | 29 | 69 |
|  | [4. Carry out practical procedures: urinary catheterisation, skin suturing] | 0 | 0 | 1 | 2.4 | 3 | 7.1 | 14 | 33.3 | 24 | 57.1 |
|  | [5. Prescribe, set up and monitor a blood transfusion] | 3 | 7.1 | 6 | 14.3 | 8 | 19 | 13 | 31 | 12 | 28.6 |
|  | [6. Prescribe dose and route of insulin, including use of sliding scales] | 3 | 7.1 | 4 | 9.5 | 14 | 33.3 | 15 | 35.7 | 6 | 14.3 |

Table 4 Current Ability to Perform Clinical Skills without Direct Supervision

|  |  | Not at all confident | | Not confident | | Unsure | | Confident | | Very confident | |
| --- | --- | --- | --- | --- | --- | --- | --- | --- | --- | --- | --- |
|  |  | n | % | n | % | n | % | n | % | n | % |
| Cuban-trained | [1. Diagnose and manage acute medical emergencies] | 0 | 0 | 1 | 3.4 | 1 | 3.4 | 12 | 41.4 | 15 | 51.7 |
|  | [2. Obstetrics: carry out a forceps delivery] | 3 | 10.3 | 2 | 6.9 | 2 | 6.9 | 10 | 34.5 | 12 | 41.4 |
|  | [3. Obstetrics: carry out a Caesarean section] | 1 | 3.4 | 2 | 6.9 | 4 | 13.8 | 5 | 17.2 | 17 | 58.6 |
|  | [4. Give an anaesthetic for minor surgery] | 0 | 0 | 1 | 3.4 | 1 | 3.4 | 11 | 37.9 | 16 | 55.2 |
|  | [5. Intubate and insert an endotracheal tube] | 1 | 3.4 | 0 | 0 | 2 | 6.9 | 12 | 41.4 | 14 | 48.3 |
|  | [6. Give health promotion advice to mothers] | 0 | 0 | 0 | 0 | 1 | 3.4 | 8 | 27.6 | 20 | 69 |
|  | [7. Conduct a health survey in a local community] | 0 | 0 | 0 | 0 | 5 | 17.2 | 10 | 34.5 | 14 | 48.3 |
|  | [8. Manage a primary health care team] | 0 | 0 | 0 | 0 | 2 | 6.9 | 9 | 31 | 18 | 62.1 |
| South African-trained | [1. Diagnose and manage acute medical emergencies] | 2 | 4.8 | 6 | 14.3 | 12 | 28.6 | 19 | 45.2 | 3 | 7.1 |
|  | [2. Obstetrics: carry out a forceps delivery] | 9 | 21.4 | 12 | 28.6 | 11 | 26.2 | 6 | 14.3 | 4 | 9.5 |
|  | [3. Obstetrics: carry out a Caesarean section] | 16 | 38.1 | 8 | 19 | 15 | 35.7 | 1 | 2.4 | 2 | 4.8 |
|  | [4. Give an anaesthetic for minor surgery] | 8 | 19 | 7 | 16.7 | 13 | 31 | 10 | 23.8 | 4 | 9.5 |
|  | [5. Intubate and insert an endotracheal tube] | 1 | 2.4 | 8 | 19 | 9 | 21.4 | 19 | 45.2 | 5 | 11.9 |
|  | [6. Give health promotion advice to mothers] | 0 | 0 | 0 | 0 | 1 | 2.4 | 22 | 52.4 | 19 | 45.2 |
|  | [7. Conduct a health survey in a local community] | 1 | 2.4 | 1 | 2.4 | 8 | 19 | 19 | 45.2 | 13 | 31 |
|  | [8. Manage a primary health care team] | 5 | 11.9 | 3 | 7.1 | 11 | 26.2 | 12 | 28.6 | 11 | 26.2 |

Table 5 Students’ Motivation for Their Choice of Medicine as a Career

| Cuban-trained students | Not important at all | | Of little importance | | Moderately important | | Important | | Extremely important | |
| --- | --- | --- | --- | --- | --- | --- | --- | --- | --- | --- |
|  | n | % | n | % | n | % | n | % | n | % |
| [1. Family wanted me to be a doctor] | 13 | 46.4 | 3 | 10.7 | 1 | 3.6 | 7 | 25 | 4 | 14.3 |
| [2. Good at sciences] | 0 | 0 | 1 | 3.4 | 4 | 13.8 | 16 | 55.2 | 8 | 27.6 |
| [3. Working for social change] | 1 | 3.4 | 0 | 0 | 5 | 17.2 | 6 | 20.7 | 17 | 58.6 |
| [4. High income potential] | 5 | 17.9 | 2 | 7.1 | 8 | 28.6 | 8 | 28.6 | 5 | 17.9 |
| [5. Desire to work in a rural/underserved area] | 1 | 3.4 | 1 | 3.4 | 4 | 13.8 | 6 | 20.7 | 17 | 58.6 |
| [6. Desire to work in another country] | 15 | 51.7 | 4 | 13.8 | 5 | 17.2 | 4 | 13.8 | 1 | 3.4 |
| [7. Social recognition or status ] | 15 | 53.6 | 6 | 21.4 | 2 | 7.1 | 3 | 10.7 | 2 | 7.1 |
| [8. Stable, secure future ] | 2 | 6.9 | 3 | 10.3 | 4 | 13.8 | 10 | 34.5 | 10 | 34.5 |
| [9. Creativity and initiative] | 0 | 0 | 1 | 3.4 | 6 | 20.7 | 11 | 37.9 | 11 | 37.9 |
| [10. Availability of jobs in the future ] | 1 | 3.4 | 2 | 6.9 | 2 | 6.9 | 13 | 44.8 | 11 | 37.9 |
| [11. Work/life balance] | 1 | 3.4 | 3 | 10.3 | 3 | 10.3 | 10 | 34.5 | 12 | 41.4 |
| [12.Could not do my preferred subject/option] | 18 | 62.1 | 2 | 6.9 | 5 | 17.2 | 2 | 6.9 | 2 | 6.9 |
| [13. Desire to help other people] | 0 | 0 | 0 | 0 | 1 | 3.4 | 7 | 24.1 | 21 | 72.4 |
| [14. Improve health in my country] | 0 | 0 | 0 | 0 | 1 | 3.4 | 7 | 24.1 | 21 | 72.4 |
| [15. Become a community leader] | 1 | 3.4 | 0 | 0 | 4 | 13.8 | 7 | 24.1 | 17 | 58.6 |

Table 5 (continued)

| South African-trained students |  |  |  |  |  |  |  |  |  |  |
| --- | --- | --- | --- | --- | --- | --- | --- | --- | --- | --- |
| [1. Family wanted me to be a doctor] | 18 | 42.9 | 9 | 21.4 | 8 | 19 | 6 | 14.3 | 1 | 2.4 |
| [2. Good at sciences] | 3 | 7.1 | 3 | 7.1 | 10 | 23.8 | 13 | 31 | 13 | 31 |
| [3. Working for social change] | 4 | 9.5 | 6 | 14.3 | 10 | 23.8 | 12 | 28.6 | 10 | 23.8 |
| [4. High income potential] | 1 | 2.4 | 9 | 21.4 | 8 | 19 | 16 | 38.1 | 8 | 19 |
| [5. Desire to work in a rural/underserved area] | 10 | 23.8 | 17 | 40.5 | 7 | 16.7 | 6 | 14.3 | 2 | 4.8 |
| [6. Desire to work in another country] | 7 | 16.7 | 14 | 33.3 | 11 | 26.2 | 5 | 11.9 | 5 | 11.9 |
| [7. Social recognition or status ] | 10 | 23.8 | 10 | 23.8 | 8 | 19 | 12 | 28.6 | 2 | 4.8 |
| [8. Stable, secure future ] | 1 | 2.4 | 4 | 9.5 | 1 | 2.4 | 12 | 28.6 | 24 | 57.1 |
| [9. Creativity and initiative] | 4 | 9.5 | 12 | 28.6 | 10 | 23.8 | 10 | 23.8 | 6 | 14.3 |
| [10. Availability of jobs in the future ] | 1 | 2.4 | 3 | 7.1 | 2 | 4.8 | 11 | 26.2 | 25 | 59.5 |
| [11. Work/life balance] | 1 | 2.4 | 7 | 16.7 | 17 | 40.5 | 9 | 21.4 | 8 | 19 |
| [12.Could not do my preferred subject/option] | 34 | 81 | 3 | 7.1 | 1 | 2.4 | 2 | 4.8 | 2 | 4.8 |
| [13. Desire to help other people] | 1 | 2.4 | 4 | 9.5 | 4 | 9.5 | 15 | 35.7 | 18 | 42.9 |
| [14. Improve health in my country] | 2 | 4.8 | 5 | 11.9 | 7 | 16.7 | 15 | 35.7 | 13 | 31 |
| [15. Become a community leader] | 5 | 11.9 | 12 | 28.6 | 7 | 16.7 | 13 | 31 | 5 | 11.9 |
